# Le Systeme Lymphatique

**Published:** 1837-10-01

**Authors:** 


### Medico-Chirurgical Review,

### No. LIV.

[No. 14 of a Decennial Series.]

JULY 1, TO OCTOBER 1, 1837.

### DESCRIPTIVE ANATOMY.

I. Le Systeme Lymphatique. Par M. *G. Breschet.* Planches 4. 8vo., Paris, 1836. Bailliére.

The Lymphatic System. By *G. Breschet.* 4 Plates.

II. Nouvelles Recherches sur la Structure de la Peau. Par M. *G. Breschet.* Planches 3. 8vo., Paris, 1835. Bailliére.

Researches on the Structure of the Skin. By M. *G. Breschet.* 3 Plates.

It is a sign of a healthy condition of the professional mind, when we observe a disposition to cultivate anatomy, and to extend the knowledge of its facts. The accurate determination of the latter is so inseparably connected with the science of medicine, that the increase of our acquaintance with them must necessarily lead to its improvement.

The diffusion of such knowledge is a great desideratum. It is not, however, very readily effected, for the majority of persons are prone to seek for the minimum, not the maximum, of attainments which will keep them afloat in their respective occupations, and far too many medical men, who have studied anatomy as a task, are apt to regard it with disgust, and to hug the idea that a very small quantum of it will serve them in the practical part of their profession.

It is not necessary for us to combat such a notion with elaborate arguments, nor to bring the heavy battery of reason against so obvious a fallacy. One consideration is sufficient of itself to overthrow it. The signs and symptoms of disease are no more than altered states or functions of the organs or the tissues:—it therefore follows as a necessary consequence, that the more intimately we understand the normal condition of those tissues and organs, the more accurately we shall understand their disordered states, the more accurately, in short, we shall understand disease.

If the man who is content to be a routinist, could calculate on always meeting with routine cases, there might be some sort of petty and contemptible rationality in his election. But, in the simplest case, contingencies may supervene, and complications, more or less obscure, may arise. Will any man of honour, will any man of prudence, voluntarily set out in life, with the determination to do just as little as possible, towards making himself master of such complications and contingencies?

If we appeal to experience, every-day experience, we must be satisfied of the de facto superiority in practice of the surgeon or physician who is thoroughly versed in anatomy. Of course we do not say that an excellent anatomist may not be a deplorable practitioner. That is not the point. The position we contend for is, that, cæteris paribus, the better the anatomist the better the practitioner, and that the reasonable cultivation of anatomy does not tend in any way to incapacitate persons for practical pursuits. We admit that men who spend all their time in the dissecting-room, who think of little else, work at nothing else—we admit that such men are conversant only with the dead, and cannot be expected to be otherwise; but that is not the kind of anatomical study we are talking of. We wish every surgeon and physician to continue to take an interest in anatomy after his collegiate examinations are over—to look at the best anatomical works—to keep himself at the level of advancing anatomical knowledge. We do not urge the high consideration, that it is pleasant to feel we are not mere laggards in science, but merely hint the more homely, but perhaps the not less potent one, that it is useful to know as much as other people.

### I. The Anatomy of the Lymphatic System.

It has been generally stated in most works on descriptive anatomy that lymphatic vessels may be discovered in every part of the human body, except in the substance of the brain, spinal marrow, eye and placenta. The recent researches however of M. Fohmann, Panizza, Arnold, and some other continental anatomists, have shewn that this assertion is not strictly correct, and that it is to be received with certain limitations. The first of these authors has described and represented with great care numerous lymphatics in the membranes and on the surface of the brain, and also those of the umbilical cord and of the placenta. Arnold says that he has detected lymphatic vessels in several of the tissues of the eye; but this announcement has not certainly been confirmed by the dissections of others; and, in the opinion of many, even the authority of M. Fohmann has not been deemed sufficient to establish the existence of lymphatics in the placenta. M. Panizza, for example, certainly one of the ablest writers on the absorbent system, confesses that he is not satisfied of the truth of this statement.

Leaving therefore these points of dispute, we shall now very briefly detail some of the most interesting and best ascertained facts, relative to the lymphatic system.

According to M. Breschet, the cellular tissue is the principal source or groundwork whence the lymphatic vessels take their origin; it is the soil, so to speak, in which their extreme roots strike and ramify.

If we observe lymphatics proceeding from, and seeming to arise out of, the substance of many organs of the body, it is because cellular tissue constitutes the basis and elementary component of these organs; and hence we find that those very parts, into which the cellular tissue does not enter, are destitute of these vessels—as, for example, the nails, horns, the epidermis, the hair, and the enamel of the teeth. Mascagni long ago announced the opinion that all the white tissues of the body are actually formed or composed of innumerable lymphatic vessels—an opinion too vague and general to be received as quite correct—and more lately M. Cruveilhier has stated in his descriptive anatomy that he deems it very probable that the cellular tissue and serous membranes primarily and chiefly consist of a meshwork of these vessels.

There are two methods in which the lymphatics of serous membranes may be exhibited—either by inserting the sharp point of the usual apparatus, used for injecting these vessels and filled with mercury, into the substance of the membrane, or by introducing some coloured liquid into the cavity lined by it, during life, or immediately after death. Those of synovial membranes may be discovered in the same manner. The lymphatics of the dermoid tissue have of late years been examined with great care by Lauth, Fohmann, and by M. Breschet himself. He has described them minutely in his work on the structure of the skin published at Paris two years ago. They are exceedingly numerous, and appear to be, for the greatest part, more superficial than the vascular capillaries. No trace however of any open orifices on the surface of the dermis can be discovered, even with a strong magnifying glass. Some anatomists indeed have stated that they have observed globules of the mercury ooze out, when pressure was made on the skin at the same time; but this phenomenon is ascribed by Breschet and others to rupture of the lymphatics, and not to simple exsudation from them. He contends therefore that there are no discoverable open extremities of the lymphatics on the surface either of the skin, or indeed of any other tissue of the body.

M. Panizza takes the same view of the subject. He details several experiments, in which he made a very successful injection of the superficial lymphatic plexus of the glans penis, and wherein, on carefully removing the epithelium, no trace of any exsuded globule of mercury could be found.

The mucous membranes, like the skin, give rise to numerous lymphatics; so numerous indeed, that they, along with minute blood-vessels and nerves, appear to constitute the villosities on their surface. Although no open orifices have ever been discovered in mucous membranes, MM. Cruveilhier and Magendie are of opinion that such do really exist.

They allude to the results of several experiments, in which the abdomen of a living sheep was opened, and milk introduced into a part of the gut. When the distended portion of the gut was compressed with the hand, the lacteals were immediately filled with the white fluid. M. Breschet however is not satisfied with the details of these experiments; and he very justly says that all forcible compression of the gut should be avoided in conducting such experiments, as it may possibly cause a rupture or laceration of the extreme ends of the tender vessels. However this may be, no person has ever *seen* the open orifices of the lacteals on the surface of the villi; and our author is therefore quite correct in condemning the practice of assuming the rationale of physiological phenomena, merely because these may be explained in a particular manner. It is exceedingly probable that imbibition, or some similar process, has something to do with the introduction of the chyle and lymph into the extreme absorbents. But in truth all that we

*know* with *certainty* is, that we *know* nothing *certain* on the subject. It remains a problem for future anatomists to solve.

Equally gratuitous is the doctrine, that the lymphatics are continuous with, and are derived from, the extreme capillary branches of the blood-vessels. It is well known to anatomists that, by pushing a fine injection into an arterial trunk, the lymphatics of the part sometimes receive a portion of the injection. This experiment has been deemed quite sufficient by many writers to establish the reality of a direct inosculation of the two sets of vessels ; nay Lippi has gone so far as even to represent such a connexion in one of his engravings.

M. Breschet however, and, we believe that we may add, almost all the best anatomists of the present day do not admit the truth of this doctrine. They attribute the passage of the injection in the above experiment to extravasation from the minute arteries, and to consecutive absorption or rather impulsion of it into the lymphatics. Our author closes his remarks on this subject by adopting the opinion of Panizza, that "anatomy has not hitherto succeeded in determining, with physical certainty, in what relation the sanguiferous and lymphatic systems stand to each other, at their extreme ramifications." If we suppose that the cellular tissue itself is in fact made up of a meshwork of lymphatic vessels, we perhaps get rid of the difficulty; but even then we cannot state, with any degree of precision, in what manner the lymph and chyle do enter into the vessels themselves. We know that the lymph and chyle contain numerous globules of considerable size, larger even than those of the blood. The question comes to be, How do those gain admission? or are we to suppose that they are developed within the tubes of the lymphatics themselves?

We thus encounter fresh difficulties at every part of the investigation; and it is therefore much wiser to confess our total ignorance of the subject.

Nothing is of greater importance in all physical researches than to acquire exactitude at every step of our investigation. Whenever we begin to frame hypotheses, unsupported by actual observation, merely for the purpose of giving a probable explanation of certain phenomena, we are almost sure to be bewildered in the mazes of our own making. We shall therefore leave the subject, on which we have been discoursing above, merely repeating our former statement that no anatomist has yet *seen* any open orifices of lymphatics in any structure of the body, either in man or in the lower tribes of animals.

We now proceed to another topic, in the history of the human lymphatic system, which has given rise to much difference of opinion. We have confessed our ignorance of the relation which exists between the capillaries of the sanguiferous and of the lymphatic systems. It now remains to be determined what knowledge we have respecting the connexion between the larger vessels of these two systems.

It has often been conjectured by anatomists, that there are some other communications between the lymphatics and the veins, in the human body, in addition to the thoracic duct, and the lymphatic trunk which proceeds from the right arm and usually terminates in the right subclavian vein. That such communications do exist in the lower classes of vertebrate animals is proved beyond doubt by the researches of Fohmann, Lauth, Panizza and Muller. They are obvious on the intestines and the mesentery in fishes, on the thigh in the frog, and on the lower extremities and the intestinal canal in birds. No such communications however are discoverable in any part of Mammals. Still the question has often been mooted whether any inosculations between the two sets of vessels, besides those above mentioned, do really exist in this class of animals.

Many of the older anatomists believed, that they had traced lymphatics to their terminations, not only in the vena cava, and v. azygos, but also in the hypogastric and lumbar veins; and, even as late as 1825, M. Lippi of Florence has maintained that the lymphatics of the digestive organs in men, as well as in other mammals and also in birds, communicate directly with the venæ cavæ, the vena portæ, the vena azygos, and with the renal and internal pudic veins. But all the best authorities, the more ancient as well as the very recent, have denied the accuracy of these statements; and it has been clearly proved that the error of Lippi has arisen from his mistaking branches of veins themselves for lymphatic vessels. Fohmann says distinctly that, during five years most laborious investigation of the absorbents, he has never detected any direct communication between the veins and the lymphatics, except in the glands, (this, we shall afterwards mention, is a doctrine not admitted by all anatomists) and in the immediate neighbourhood of the termination of the thoracic duct.

But although no communication can be traced with the larger veins, some authors have contended that the lymphatics inosculate directly with the smaller venous branches in different parts, and especially with those of the mesentery.

M. M. Fohmann and Panizza however expressly assert that they have in no instance been able to detect such a connexion.

The only other hypothesis on the subject of venous and lymphatic communication, which remains to be mentioned, is that which supposes that the two sets of vessels inosculate with each other in the substance of the absorbent glands. This opinion was adopted by the first Meckel, and also by Caldani.

Monro Secundus and Mascagni were opposed to it; but, of recent years, it has been again very ably advocated by Fohmann, Tiedemann, Lauth and Panizza.

It has been long known that, when mercury is injected into those lymphatics which proceed on to a gland (vasa inferentia), part of it passes more or less freely into the neighbouring veins, as well as into the lymphatics leading out of the gland (vasa efferentia). This transmission is sometimes so very free, that it is actually necessary to put a ligature around the veins, in order that the lymphatics may be well filled.

Various explanations have been given of this occurrence. Some writers have supposed that it was always the result of rupture of the glandular tissue; others have attributed it to a direct communication between the two sets of vessels; and lastly it has been conjectured that it might be owing to a mere transudation from one set of vessels to another.

It cannot indeed be doubted that, in not a few of the experiments, there has been an actual rupture of the substance of the gland, and that the quicksilver has escaped into the cellular substance, and been forced into the lacerated vessels. But this has certainly not been the case in all. The greatest delicacy has been used by the anatomists last mentioned to avoid all force or compression in their experiments; and we cannot therefore fairly presume that extravasation of the mercury has always taken place. No traces of such extravasation have been observed; and moreover it has been often remarked that the veins were filled with the quicksilver even before the lymphatics, which issued from the gland, were injected. It may also be added, that, if an extravasation had occurred, we might reasonably expect that the small arteries as well as the veins should receive a portion of the injection; and yet this is never the case. M. Fohmann considers these arguments quite conclusive; and he has supported with great ability the doctrine, that there is in truth a direct communication between the lymphatics and veins of an absorbent gland. It must however be admitted that, although there are some very plausible arguments in favour of this supposition, no direct observations can be adduced in its support. M. Panizza has mentioned one fact which seems to be very unfavourable to it. He says that he has never succeeded in injecting the lymphatics, when he reversed the mode of performing the experiment—viz. that, when the mercury was injected by the veins, none ever entered the lymphatics. This result might certainly have been expected, if there is really so direct a communication between the two sets of vessels, as M. Fohmann maintains.

Panizza therefore and Muller also are inclined to believe that the passage of the mercury, in the substance of the absorbent glands, from the lymphatics into the veins, is probably effected by means of minute pores in the sides of the vessels, analogous to those which permit the atmospheric air to act upon the blood in the pulmonary cells.

Breschet however, without giving a decided opinion, appears to lean to M. Fohmann's hypothesis that there is a direct communication between the lymphatics and the minute veins within the substance of the absorbent glands—a doctrine that receives considerable support from the results of comparative anatomy, and also from the carefully performed experiments recorded by Meckel, Fohmann, and many others.

Whatever view we take of this subject, there is every reason to believe that during life fluids may pass somehow or other from the lymphatics into the veins of the glands. If we admit this doctrine, we can readily understand in what manner certain substances may pass very quickly from the stomach and bowels into the mass of circulating blood, and be appreciable at different emunctories, within a very short time after they have been introduced into the alimentary canal.

We now proceed to examine the development of the lymphatic vessels in the different classes of animals; and we shall here find that a knowledge of comparative anatomy is quite necessary to a correct understanding of the organization and functions of the human body.

The absorbent system, as indeed all the other systems of the body, is more extensively developed and more complicated in the higher animals, than in those lower in the scale. In man it reaches its maximum of development. We shall briefly review the structure of this system in the different orders of vertebrate animals; premising that no very distinct traces of its existence have been discovered in any of the invertebrate class.

*In Fishes*, the lymphatics were discovered and very accurately described by Dr. Monro 2ndus, and by Mr. Hewson. The study of this branch of comparative anatomy has of late years been admirably prosecuted by M. Fohmann, whose work (Das Saugader-system der Weichthiere, 1827) is illustrated by most beautiful engravings. The lymphatics of fishes have no valves, nor are there any glands connected with them. They form numerous plexuses; the most important of which is situated between the mucous and muscular coats of the intestinal canal, and terminate in a large reservoir placed at the right side of the cardia. From this point proceed other plexuses, which at length reach and open into the jugular vein. At numerous other points, however, a communication may be readily traced between the lymphatic and venous systems. The absence of lymphatic glands in fishes seems to be compensated for by some remarkable dilatations of the vessels themselves, in the middle of those meshes or plexuses, which they form in different parts.

In *Reptiles* also we cannot discover any lymphatic glands; but the vessels are certainly provided with valves—few however, and so incomplete that they do not prevent injections to pass freely from the trunks into the smaller branches.

According to Carus and others, the lymphatics of the inferior region of the body unite in a common reservoir, whence proceeds a double plexus, which then forms a sort of membranous sheath around the descending aorta, and, communicating superiorly with the plexus of the neck, terminates at length in the two jugular veins. What is most remarkable in this class of animals is the existence of pulsatory vesicles, or, as it were, hearts, along the course of the lymphatic vessels. This very curious discovery was made, about nearly the same time, by Muller and Panizza. Muller found in frogs and toads two pairs; one under the skin in the sciatic region, and the other more deeply situated upon the third cervical vertebra. Their pulsations, independent of those of the heart, do not seem to be at all isochronous. The inferior set discharge their lymph into a branch of the sciatic vein, and the upper set into a branch of the jugular vein.

Panizza has discovered these pulsating lymphatic vesicles in several of the serpent tribe. He found that they exhibited in the living animal an alternate expansion and contraction, analogous to the diastole and systole of the heart; and that the pulsations were not synchronous either with those of this organ, or with the movements of respiration. If they were pricked during their contraction, a reddish fluid was observed to flow out. When the vein, with which one of these vesicles communicated, was obstructed, it (the vesicle) remained distended. It was quite evident that the pulsatory movements of these lymphatic vesicles were independent of the action of the heart or arteries, as they were found to continue after the extirpation of the heart and the division of the arterial trunks.

The lymphatic system in *Birds* was discovered by Hunter, and has since his time been examined with great care by Hewson, Tiedemann and Lauth. The valves of their lymphatics are very imperfect; but in this class of animals we first discover rudiments of glands along the course of the vessels in the neck, and also at the basis of the ventral aorta.

According to Tiedemann, these glands are more developed in aquatic than in terrestrial birds. Magendie has well described and figured those of the neck. In other parts of the body, we find, in place of absorbent glands, plexuses of vessels; the most considerable of which, situated near the cœliac artery, gives off two canals. These terminate in the subclavian veins. Besides this communication of the lymphatics with the veins, there are several others, equally obvious to the naked eye, between these two systems.

In the class of the *Mammifera,* the lymphatic system acquires its highest degree of development and complexity. The trunks of the vessels are more numerous; their valves are more perfect; the plexuses are less in number; the glands are very much more frequent; and the communications—at least the visible ones—with the veins are reduced to a few points. Another peculiarity also is that the lymphatics are almost always arranged in two sets—the one superficial, the other deep-seated.

The lymphatic trunks are generally larger and more distinct in the lower Mammifera, than in man; and the absorbent glands are proportionally smaller. The mesenteric glands are much more detached from each other in all those animals whose intestines are very long, than in those whose intestines are very short. In the Cetacca, these glands present certain peculiarities which deserve to be noticed. The late Mr. Abernethy, in a paper printed in the Philosophical Transactions for 1796, has stated that, by injecting the mesenteric arteries, he discovered that a communication exists between these vessels and certain cavities in the middle of the mesenteric glands; and he also stated that the mercury passed from the lacteals into these glandular cavities. He says moreover that the cavities themselves communicate with each other, by means of lymphatic vessels.

M. Breschet, however, is not inclined to believe in the accuracy of these statements, although he confesses that he is not prepared to refute them by any observations of his own.

He sums up his description of the lymphatic system of the Mammifera, by the following general remark.

"In proportion as the animal organization becomes more complicated, the lymphatic system is perfected, and acquires, so to speak, a more independent existence; it gradually loses the cellular and plexiform appearance, and assumes that of a multitude of genuine tubes or canals; its valves develope themselves; its glands make their appearance and become gradually more large and numerous; finally, its connexions with the venous system are much fewer, and at length in men are reduced to those which are indispensable for the transmission of the lymph into the torrent of the circulation."

Having thus shortly described the anatomy of the lymphatic system in the several classes of Vertebrate animals, we shall proceed to offer a few remarks on the functions which it is supposed to perform in the animal economy.

It is unnecessary to allude particularly to the opinions held by Swammerdam, Boerhave, Monro, and other anatomists, even after the discovery of the lymphatics, that the function of absorption was performed exclusively by the veins. The reasonings, which they adduced, were founded on false premises, and they cannot therefore much influence our speculations.

It is to the labours of the two Hunters that we are chiefly indebted for having proved, beyond the reach of doubt, that the chyle is taken up by the intestinal absorbents, and conveyed by them into the general mass of the circulation. Dr. William Hunter indeed was inclined, for many years afterwards, to admit the co-operation of the veins in this important operation of life; but his younger brother, endowed with a bolder and more independent mind, maintained that this opinion was quite inaccurate. By a series of well conceived experiments, he quite satisfied himself that the absorption of the chyle is performed exclusively by the lacteals. Having opened the abdomen of a living dog, and discovered those points where the lacteals were full of milky chyle and others where the chyle was yet transparent, he took two portions of the intestine, and passed ligatures around each, after having tied the branches of the mesenteric arteries and veins which supplied them. He then introduced some milk into each portion. The lacteals of one of these continued to transmit a white fluid; and those of the other, which hitherto had been nearly pellucid, began to be filled with a similarly opaque fluid, while the blood drawn from the veins presented no traces of milk.

This experiment was repeated several times without tying the veins; and still the same results were uniformly obtained.

In a sheep, which had not taken food for several hours, the abdomen was opened. The lacteals were found full of a limpid transparent fluid. A solution of isinglass, coloured blue, was then injected into several folds of intestine; and these were then secured by ligatures at two points. Being replaced in the abdomen, and then examined some time afterwards, the lacteals presented a blue appearance; but the blood, which was drawn from the veins of the parts, exhibited no discolouration, even after it was permitted to coagulate.

These and similar experiments were repeated with various modifications by Hewson, Shelden, and Cruickshank; and the result of all was, that it was universally admitted as an established fact in physiology, that intestinal absorption was performed solely and exclusively by the lacteals.

Hitherto the attention of anatomists had been almost entirely directed to intestinal absorption; and induction only had led to the belief that the general absorbents of the body exercised a similarly exclusive function in other parts. Schreger indeed had attempted to prove the truth of this by direct experiments, wherein he exposed the limbs to various coloured and odoriferous solutions, and which in his opinion shewed that the substances were invariably absorbed by the lymphatics and never by the veins.

But as the facts which he states, and the conclusions which he draws from them are contradicted by the experience of some of the ablest anatomists of the present day, it is not worth while to narrate them. It is to be observed, however, that they obtained such universal credit, that they induced the distinguished Mascagni and others to admit their unexceptionable accuracy. M. Lauth, one of the ablest physiologists of the present Strasbourg School of Medicine, has given new force to them, by stating that injections of ink into the thoracic cavities of dogs, recently dead, are always followed by the appearance of detached dark spots, or of distinct and separate absorbent trunks filled with dark fluid, on the surface of the pleuræ.

For many years the doctrine, that the lymphatics were the sole and exclusive vessels for absorption, was so undisputed, that it seemed that physiologists were scarcely aware that any other opinion had ever been held by their predecessors. Such was the state of opinion till the year 1809, when Magendie read to the French Institute his celebrated Memoir to prove that "the lymphatic vessels are not the only route by which foreign matters are admitted into the sanguiferous circulation." We shall briefly allude to a few of his experiments. A portion of gut in a living dog was tied, and then detached from all its connexions, save only from a single mesenteric artery and vein by which it hung suspended. A small quantity of the Upas poison was then injected into its cavity, and the portion of the gut was then replaced in the abdomen. At the end of six minutes, the general effects of the poison had developed themselves in their full intensity, just as if the bowel had still retained all its ordinary connexions.

The thigh of a dog was detached from the trunk, leaving only the crural artery and vein undivided. These vessels were then opened, and a crow-quill was introduced into their tubes: they were next secured around the quills, each by two ligatures; and their coats were afterwards cut across between these. The only communication therefore now between the body and the severed limb was through the medium of the arterial and venous blood passing along the quills. When the Upas was inserted into the foot of the animal, its effects were observed to take place as speedily as if the limb had sustained no dismemberment. This experiment has been repeated by two German physiologists, Emmert and Rapp, and by several others, with the same results.

M. Westrumb found that, when he introduced a solution of ferro-cyanate of potassa into the stomach, traces of the salt were discoverable in the urine,* although none could be detected in the lymph or chyle. M. Foderé performed a similar experiment on a portion of gut; and found, he says, that the mesenteric veins, as well as the lacteals, of the parts exhibited a blue colour, when the gut was inserted into a solution of sulphate of iron; but M. Kolk denies the accuracy of this assertion, and says that in his experiment the blue colour was observable only in the lacteals.

It would occupy far too much space to detail the opinions of all the authors, who have written on this subject since the date of Magendie's Memoir. Suffice it to say that M. M. Tiedemann and Gmelin, two of the most accurate enquirers of the present time, admit that many foreign substances, introduced into the intestines, are discoverable in the urine and also in the blood, although no traces of them are to be met with in the absorbents.

"If," says M. Breschet, "after all the facts now detailed, we must admit that the lymphatics seem to have very little action upon substances introduced

---

* The extreme rapidity, with which the transmission of soluble substances takes place through membranes, is well exemplified in the following experiment related by Muller in his Physiology.

He took a small flask filled with a solution of the cyanuret of potassa, and covered its mouth with a frog's bladder; he then very lightly wetted it with a solution of sulphate of iron, and immediately inverted the phial. A blue colour was perceptible in the course of a *second*.

Now a frog's bladder is very much thicker and less permeable than the villous coat of the intestines, which Muller estimates to be of extreme tenuity—not exceeding in thickness the ·00174 of an inch. If we suppose that the complete circuit of the circulation is performed in two or three minutes, it is obvious that we are at once furnished with a rational interpretation of the great rapidity with which certain substances may be conveyed from the intestines to the remotest parts of the body, without having recourse to the old notion, recently revived by Sir E. Home and Lippi, that there are any direct channels between the alimentary tube and the urinary bladder.

from without (the chyle excepted), they most unquestionably exercise such a power over those which are generated in the body. The bile, for example, may be absorbed by them—a fact indisputably proved by the researches of the two physiologists named above."

M. M. Foderé and Lauth have met with blood in absorbents; Magendie and a host of other physiologists have found purulent matter in their cavities; Sir A. Cooper discovered a white cerebriform substance in the thoracic duct of a man, who died of fungus hæmatodes of the testicle.

In concluding his review of the conflicting opinions on the subject of venous and lymphatic absorption, our author adds that the solution of the problem is perhaps less obscure, than has often been supposed. He supposes that the veins and absorbents communicate with each other in the substance of the glands, as they are seen to do in some of the lower vertebrate animals in various parts of the body. The chief point of doubt is, as to the manner in which this communication is effected. M. Breschet does not think that it can be by direct communication with each other by open mouths; but rather "par une force absorbante, analogue à celle dont jouissent les radicules lymphatiques, que les veines doivent absorber le contenu de ces derniers vaisseaux dans les glandes."*

In fine, he supposes that the veins of every part have the power of absorption, as well as the lymphatics; but he is unable to specify the limits and the circumstances in which each set of vessels exercises this function.

He alludes to a question of great obscurity, and which in all probability will always elude the search of our best-directed enquiries—viz. how and in what part are the globules of the chyle formed? We cannot suppose that they are already formed before the chyle is absorbed from the food, as they are known to be of considerable volume, and as no open mouths of the lacteals have ever been discovered in the villi of the intestines, even with the aid of the strongest magnifying glass. It is an utter loss of time to form mere idle conjectures on this subject. "Ne craignons pas de dire," says M. Breschet, "que l'on ignore completement!" There are many other points in the anatomy and physiology of the absorbent system, which are little, if at all, accurately understood; and we are therefore compelled to confess that we know but little, and that little only imperfectly, of one of the most important functions of animal life.

The following statement of the chief conclusions, to be drawn from the preceding remarks, may be useful to the reader.

1. In no part of the body have any open extremities or orifices of absorbents been discovered.

2. No direct communication between the arterial or venous capillaries and the absorbent vessels has been distinctly proved to exist.

3. Fohmann and some other anatomists assert that a communication exists between the absorbent vessels and the veins, within the substance of the alveolar glands. This doctrine, although probable, is not admitted by all.

---

* The doctrine—that there is a free communication between the veins and the lymphatics in the substance of the absorbent glands—is maintained by that admirable anatomist, M. Fohmann; and certainly the comparative anatomy of the lymphatic system, as we have described above, affords considerable shew of confirmation to it.

4. The absorbents in fishes are not provided with valves, or with glands. They communicate freely, in different parts of the body, with the adjacent larger veins.

5. In reptiles the lymphatics have imperfect valves; but there are no glands. We discover however along their course certain dilatations which are seen during life to pulsate, and which appear to force the lymph into the adjacent veins.

6. Rudiments of absorbent glands are discoverable in birds ; and in them there are several other direct communications between the lymphatics and the veins, besides the chief one at the root of the neck.

7. In mammals, the lymphatic system is more complicated; the valves are more numerous and more perfect ; the glands are more numerous ; and the communications between the lymphatic vessels and the veins appear to be fewer in number and much less direct.

8. The veins seem to have the power of absorbing foreign substances admitted into the body, as well as the lymphatics ; but it is still doubtful whether they ever absorb chyle or any other fluid generated within the system.

### II. The Anatomy of the Skin.

The next of the works upon our list is still from the pen of M. Breschet, able anatomist—and on the structure of the skin, an important subject. Unfortunately its difficulty is commensurate with its importance, and the discrepancies of observers have corresponded with its difficulties.

M. Breschet, and his colleague in the work, M. Roussel de Vauzème, have used all the means in their power for arriving at the truth. Time has been consumed and labour expended on an inquiry which all must acknowledge to be intricate, and which they confess to be on their part incomplete. They deprecate criticism, indeed they may be said to defy it, for they protest in limine that they will pay attention to no strictures but to those which emanate from persons who have made the structure of the skin a subject of their special investigation. This is a bold, but perhaps it is not altogether an unexceptionable determination.

They treat their subject in the following order. They first give a summary of the constituent parts of the skin, and then examine each in detail.*

### 1. Constituent Parts of the Skin.

1. The *Derma* (cutis vera—corium—true skin)—a dense, fibrous, cellular web, enveloping and protecting the sanguineous capillaries, the lymphatics, the nerves, and the other organs contained in the skin.

2. The *Papillæ*—the organs of touch, in which the nerves are finally dis-

---

* We shall chiefly select such facts, or such opinions for notice, as possess some claim to novelty, or are otherwise important. We must presume that our readers have read Beclard's, or Craigie's, or some other work, which contains what is generally known or believed in regard to the cutaneous tissue.

tributed. They are small nipple-like projections, slightly curved, their apices blunted, and concealed beneath several envelopes.

3. The *Diapnogenous Apparatus,* (the organs of the secretion and excretion of the sweat—from διαπνοὴ, transpiratio, perspiratio)—is composed of a glandular parenchyma, and of *sudoriferous* or *hydrophorous* canals.*

The parenchymatous or secreting organ is enclosed in the derma, and gives origin to the excreting canals; the latter are of a spiral form, pass between the papillæ, and open obliquely on the external surface of the epidermis.

4. *Apparatus of Inhalation, or Absorbent Canals.* These resemble, in many respects, the lymphatic vessels. They are situated in the corpus mucosum, which forms the most external layer of the skin, for the cuticle or epidermis is only a dependence of the mucous body. The inhalent canals appear to be unprovided with mouths or with absorbent openings. They commence in the most superficial layer of the cuticle, but their mode of origin is very difficult to be determined. By their other extremity they communicate with a net-work of vessels, which our authors believe to be lymphatics mixed with veins.

5. *The organs which form the Mucous Matter, or the Blennogenous Apparatus.*† This is composed of a glandular parenchyma, or organ of secretion, situated in the thickness of the derma—and of excreting canals, which issue from the preceding organ, and deposit the mucous matter between the papillæ.

6. *Chromatogenous Apparatus, or that for producing the Colouring Matter.* This is composed of a glandular or secreting parenchyma, situated a little below the papillæ, and presenting particular excreting canals, which pour out on the surface of the derma the colouring principle. This mingles with the soft and diffluent mucous matter, and it is their mixture that produces the "pretended reticular body of Malpighi," and the epidermis or cuticle. We must attribute to this double apparatus the production of horns, scales, prickles, hairs, nails, hoofs, and so forth.

Our authors have ascertained, that of all the cutaneous expansion in man, the skin of the heel is the best adapted for anatomical examination, on account of the thickness of the derma and the mucous matter. To this part, therefore, their observations must be understood to apply. The anatomical causes of the difference displayed by different portions of the skin will be discussed at a future opportunity.

Such is the sort of table given by our authors of the anatomical elements which constitute the skin. The enumeration will at once convey to the well-informed anatomist the essential differences which exist between it and the ordinary descriptions of the cutaneous apparatus. On this point we need not dwell, but proceed to display the opinions of our authors in detail.

The body of the work is divided into six chapters. The first is on the derma—the second on the papillæ—the third on the diapnogenous apparatus—the fourth on the apparatus of inhalation—the fifth on the blennogenous apparatus, or organs which secrete the mucous and the horny matter—the

---

sixth on the chromatogenous apparatus, or organs for the secretion and excretion of the colouring matter. Appended to these chapters are some remarks on the pathology of the skin, and some conclusions.

### I. On the Derma.

On this head our authors say little that is new; and we shall confine ourselves to stating that the result of their observations amounts to this—that the derma is a membrane, the fibres of which, firmly interlaced with one another, leave interstices, areolæ, or cellules, which protect and insulate a great number of organs. On its external surface it insensibly merges into a membrane, which appears to be confounded with the parenchyma of the colouring and papillary organs. The derma, according to our authors, is by no means the inextricable net-work of fibrous tissue which some authors have represented it to be. It contains and supports organs, the precise description of which will come by-and-bye.

In serpents, the derma has a remarkable disposition: it is raised into imbricated prominences, covered by a thin layer of epidermis, and their whole constitutes the scales. In fish, on the contrary, the surface of the derma is uniform, and the scales are entirely composed of horny matter.

### II. On the Neurothelic Apparatus, or the Papillary Bodies.

There is no doubt that the filaments proceeding from the different nervous trunks in the subcutaneous cellular tissue divide to extreme minuteness on approaching the derma. However carefully we endeavour to dissect them, we most commonly lose them in the structure of the derma, by reason of *its* opacity and *their* delicacy. The only chance of distinguishing them, in the midst of the vascular net-work of the derma and of its excretory canals, is to observe very carefully the point where they enter and to which they tend, and with practice and dexterity the nervous filaments may be picked out as they approach the mucous body, where they are delicate, almost pulpy, and penetrate the base of the papillæ.

The papillæ themselves are ranged in rows, generally bifid or trifid, separated transversely by the interval in which the sudoriferous canals are lodged, and longitudinally by the grooves from which the horny or epidermic matter proceeds.* Their form is that of a small cone, the base of which is lost in the derma, while the summit ends in a blunt point. Each papilla penetrates the horny matter, as a sword enters its sheath, and the internal surface of the epidermis exactly represents, by its symmetrical depressions, the number and disposition of the papillæ.

The attachment of the papillæ to the derma is firmer than that to the

* The terms "mucous matter," "horny matter," are used almost indiscriminately, because the horny epidermis is supposed to be formed by a kind of inspissation of the external surface of the mucous matter. We beg our readers to bear this in mind when they find the term "horny matter" occur.

epidermis, so that if a little force is used, the papillæ separate from the latter, but remain in connexion with the former.

The direction of the papillæ in the epidermis is oblique, and slightly inclined. Besides a sheath which they get from the derma, the horny matter supplies them also with a sheath, which covers them in the manner of a hood. This is very dense in the heel. The summit of the papillæ is not perforated by any opening.

Our authors refer to the structure of the papillæ in the whale—which they say resemble those of man so closely as to make the same description applicable to both—as exhibiting their form and structure on an intelligible scale.

The papillæ of the whale, then, spring from the derma in innumerable quantities, piercing the horny matter like a sieve, or rather making it resemble a series of organ pipes, perforated to give them passage.

Their length varies with the thickness of the skin in the different regions of the same animal, or in animals of different species.

The nervous twigs, though united sometimes in twos and threes on a common base, are always contained in a particular sheath furnished by the horny matter which is moulded on them. In its thickest part, the body of the nerve presents across the neurilema slight undulated striæ, which proceed from the base, become less distinct, indeed become confused, and wind to a kind of button-like termination, where they appear to unite in concentric semi-circles. This surface is smooth and uniform, and has no communication with neighbouring parts. Examination under the microscope, or in any other manner, discovers in the papillæ a white dense tissue, more easily torn than broken, analogous in all respects to the nervous. It is not possible to separate into fasciculi the striæ or undulated lines apparent on the exterior; but our authors have distinguished the opening of a nutritious vessel in the interior. In the papillæ of man they have found two, which appear to unite and form an arch. These vessels are distinctly perceived, when the papillæ are injected, and divided by a transverse section. Besides this, in the centre of the neurilema or sheath, there appears to be a whitish pulpy substance.

From these circumstances discoverable in the papillæ of the whale, our authors think that it is not possible to entertain a doubt with respect either to the structure or the sensorial functions of these bodies.

The papillæ of the tongue are usually considered as peculiarly adapted for the sense of taste, and, consequently, as essentially nervous. But if the papillæ of the tongue of the ox be carefully examined, it will be found that they are enclosed in a horny case of greater or less thickness, which must be opposed to the exercise of such a function as that of taste. In the interstices, however, between the papillæ, there exist below a very fine epithelion true nervous papillæ *(tiges nerveuses)*, similar, or nearly similar, to those which have been already described. The number of these latter is considerable, and it is in them, according to our authors, that the sense of taste exclusively resides. The common papillæ of the tongue are thought by them to be rather devoted to the function of touch, and even to that of triturating the finer molecules of the alimentary mass, and to render it, in consequence, of more easy appreciation by the papillæ of taste in their intervals. On this point our authors have not made up their minds, but intend to examine it with more attention hereafter.

On the whole, our authors conclude that it would be difficult to form any opinion but the one they have advanced on the nature of the tactile organ. On the surface of the derma there are elevations and intervening furrows. The former correspond to the nerves; the latter to the sudoriferous and inhalent canals, while the source of the horny matter is at their bottom. If the papillæ are not the organs of touch in the skin, what are?

The mode of termination of the nerves occupies the attention of our authors. We see the nerves under three points of view:—1, in the subcutaneous cellular tissue they have the usual characters of spinal nerves; 2, in the substance of the derma they become soft, flexuous, and capillary; 3, on the external surface of the derma they are transformed into symmetrical papillæ.

The question—What becomes of the neurilema of the nerves, on their entrance into the derma? naturally forms a subject of enquiry. Our authors suppose that the nerves become divested of it, and that it becomes blended with the derma. It is probable, however, that some sort of envelope still invests the nervous matter. What it is, if any, is dubious, nor is it necessary to follow our authors through their analogical speculations on the subject.

The essential organ of touch, then, consists of—

A. *A principal part*—the nerve of touch, terminating in a blunt point.

B. *Of accessory and protecting parts*—1, the derma, enclosing the nerve in its interior; 2, the papillary neurilema, furnished by the derma; 3, of a proper sheath, of peculiar and horny tissue; 4, of a fine layer of epidermis, covering the sheath, and indispensable for the exercise of the function of touch.

If any one of these conditions should be absent, or modified in a certain manner, the sense of touch cannot be exerted. It is evident that the derma, the dermic sheath, the proper sheath of epidermis, are to the nerves of touch what the complicated apparatuses of sight and hearing are to the optic and acoustic nerves.

If we compare the senses of taste and smelling with that of touch, we must perceive a greater delicacy in the apparatus of the former, and a predominance of the horny covering for the nervous terminations and expansions in the latter. Touch, too, is exercised by nervous bundles, enclosed in a tissue which insulates each, whilst the nerves of sight and hearing are expanded at their termination.

Our authors examine the hypotheses which are entertained with regard to the ultimate disposition of the nerves. We need not go into a subject so intricate, and, we may add, so unsatisfactory. Their observations on the papillæ bear out, in some measure, the conclusions of MM. Prévost and Dumas, who have conceived that nerves end in a loop. In the papillæ, the microscope, if it may be trusted, represents the nerves as preserving their filamentous disposition till they arrive at the top of the papilla with its horny covering, where they form concentric loops.

### III. On the Diapnogenous Apparatus, and on the Sudoriferous or Hidrophorous Canals.

This exhalent apparatus occupies the thickness of the derma, and extends from its interior to the most superficial layer of the epidermis, where it presents an opening.

It is composed of a secreting parenchyma and of an excretory duct.

The parenchyma is situated in the thickness of the derma, and is surrounded with numerous capillaries attached to it. The form is that of a sac slightly swollen, from which issues a spiroid duct, which pursues its course in the derma, and issues from it by the infundibulum, or transverse fissure, situated between the papillæ; the duct then proceeds obliquely through the horny substance, in a corkscrew form, to without the epidermis, where its termination is indicated by a slight depression, or kind of pore, which may be remarked on the prominent epidermic lines.

The duct, as it passes through the epidermis, is rounded. The structure closely resembles that of the horny tissue, from which it is difficult to distinguish it. Its spiral form causes it to open externally by a very oblique opening, almost parallel to the plane of the skin; the opening itself closing by the mutual application of the superior and inferior walls of the tube. If we look at a drop of sweat as it exsudes, we shall see it preceded by an elevation of the epidermis, like a pump-valve.

If a piece of skin be macerated, so that the epidermis can be separated from the derma, we may see, with the naked eye, these excretory ducts indefinitely lengthen, like threads of spider's web. These are the spirals unrolled. When examined by the microscope, they present a surface covered with horny matter, which seems disposed in an imbricated manner on a central canal. We may distinguish very well, in this way, the issue of these canals from between the papillæ, and their penetration into the horny matter, which fills the funnel-like depressions between the papillæ. At their exit from the derma, the spiral tubes are accompanied by an inhalent vessel, which enters into the infundibular depression. The spiral, filiform tubes, when taken up with a pair of forceps, and placed upon a moistened piece of glass, roll up, and form a sort of homogeneous mucous mass, elastic and trembling like a piece of jelly. On stirring them, there are detached a great number of irregular polygonal scales. The sweat found in the secreting organ issues through the sinuosities of the excretory duct, which must also give exit to the insensible perspiration. Probably, say our authors, were we to watch the external orifice of one of these ducts when the body is heated in cold weather, we should find it smoke like the pipe of a stove.

This corkscrew disposition of the sudoriferous ducts in man, explains the anomaly of the epidermis transmitting the excretions, and yet appearing imperforate when examined separately. If the membrane is raised in the living body or the dead, the sudoriferous tubes are torn from the derma, retract, and close the opening in the epidermis, the spiral coiling on itself. If the epidermis is detached in several layers, each contains a fragment of the spiral tube, with its two openings, lying almost parallel with the plane of the layer, and not corresponding with one another. The walls of the tube come into apposition, and in this case, as in that of the entire epidermis, an opening is no longer discoverable.

If the horny layer be sliced off, and if, before arriving at the papillæ, we squeeze the skin between the fingers, as we squeeze a piece of orange-peel, we may perceive drops distilling from the pores, which correspond to the infundibuliform depressions between the papillæ, that is, to the situation of the sudoriferous canals.

Another experiment is mentioned by our authors, as evidence of the tubular nature of the spirals. If a hole is made in the horny matter of the heel, in a direction parallel to the plane of the skin, and if a little mercury is poured into it, and if then a thin slice of the exterior of the epidermis is taken off with a razor, and the mercury pressed over with the handle of the scalpel, the metal may be seen issuing by all the sudatory canals in the spot. We see the same thing in the palm of the hand and end of the fingers, when the sweat oozes from them.

Our authors have carefully examined the epidermis of the whale, in order to discover how liquids traverse it from within. Pressure of the fluid out of the sudoriferous tubes, always occasioned the elevation of a little valve of epidermis before it escaped externally. Our authors insist upon these facts, because, although exhalent vessels have been talked of, they have not been sufficiently described nor satisfactorily demonstrated.

After stating and commenting on the various opinions that have been expressed in reference to the sudatory canals, our authors remark that the recent observations of Eickhorn are among the most interesting and most satisfactory. Yet he has committed several mistakes. He believes them, for example, conical, with an infundibuliform opening, and of sufficient diameter to admit of the introduction of a hair.

Our authors themselves allow that it is extremely difficult to ascertain the exact disposition of the sudatory canals, and that it has cost them much time and trouble to obtain the knowledge they have communicated on the subject. It is possible that they may hereafter be proved to be in error; but mere criticism, without a careful examination of the skin, and a repetition of experiments, will be insufficient to do so.

### IV. ON THE CUTANEOUS APPARATUS OF INHALATION.

In order to examine this most satisfactorily, we must remove a thin slice of the epidermis,* taking care to select a soft and white portion. The slice which has been removed is to be placed on a piece of glass with a few drops of water, and after we have satisfied ourselves that there is no extraneous substance along with it, we may proceed to dissect it with curve-pointed instruments.

---

* Our authors consider all external to the derma as epidermis. The most external layer of it, which we are here directed to remove, is the cuticle, or epidermis of most authors. When the most superficial layer of the epidermis is mentioned, *the* epidermis, of common parlance, is meant.

The inhalent canals appear to be situated below the most superficial layer of the epidermis. They present the form of separate radicles spread out in the horny tissue, where they anastomose several times, and then penetrate the derma by the infundibulum of the papillæ, near the sudoriferous canals. All these vascular trunks, symmetrically arranged in the fissures between the papillæ, which they traverse, communicate in the derma below the papillæ with canals, forming a common plexus. Our authors confess that they have seen this termination of the inhalent canals very seldom; yet they feel confident that they *have* seen it.

These vessels are of extreme tenuity, and ramify in the form of loops in a hard, elastic, resisting substance. They themselves break with great facility. When examined in the microscope, their colour is white and silvery, and they are crossed by diaphragmatic septa ; a circumstance which establishes their analogy with the veins and the lymphatics. Sometimes they are knotty, at others they are smooth. They may be distinguished with a very weak lens, and even with the naked eye, on scraping the surface of the epidermis. They are occasionally long, and resemble very fine hairs.

In order to perceive the entrance of the inhalent vessels into the derma, we must lightly raise the epidermis. We may then, with the assistance of a magnifier, observe all the hidrophorous canals, accompanied with an inhalent vessel, the two being very intimately united near the derma, although they speedily separate.

With the microscope we may distinguish the difference between the two sets of vessels. The sudoriferous canal is the larger, covered with small imbricated laminæ, serpentine in form, and elastic. The inhalent vessel is smooth, silvery, straight or slightly curved, and traversed by a visible central canal, imperfectly interrupted by small septa. If the epidermis is separated too violently from the derma, the inhalent vessels are broken, and only the sudoriferous canals, which are capable of considerable elongation, remain. Another mark of distinction is this:—the inhalent vessels have anastomotic ramifications, and a plexiform arrangement, which the sudoriferous canals have not.

Our authors mention an experiment which appears to them to indicate the existence of a direct communication between the vascular capillary system and the inhalent canals. If an injection is thrown into the main artery of a limb, it always stops at the derma. If, then, the skin is removed in thin slices, while pressure is made with a scalpel on the part that has been injected, the inhalent canals may be seen coloured in the horny tissue, as they ramify and anastomose beneath the most superficial epidermic layer. The sudoriferous and inhalent vessels cannot be dissected throughout their entire extent, on account of the resistance offered by the horny tissue, but the one may be seen in fragments by the microscope, and the other entirely by means of injections.

Whatever may be the colour of the horny tissue, the absorbent canals, the nerves, and the sudoriferous canals are always white.

Our authors have been unable to discover external orifices to these inhalent canals. They are disposed to think that fluids are first imbibed by the horny tissue, and then absorbed by the inhalents. Our anatomical readers need not be reminded of the contradictory statements that have been made with respect to the orifices of the lacteal vessels. The difficulty of distinguishing the apertures of the cutaneous inhalents (should there be any), must be very much greater. Our authors engage deeply in the examination of opinions on the nature of the intestinal villosities, and of the lacteal apertures. But we need not follow them.

We may mention, however, the method of injecting the lymphatics of the skin adopted and recommended by our authors. Sometimes they have introduced the mercurial tube into a lymphatic vessel of the leg, and the mercury has run into the cutaneous lymphatics of the groin. This plan frequently fails. In other instances they have introduced the mercurial tube directly into the cutaneous tissue, at the point where they desire to examine the lymphatics, and to be certain of not mistaking the sanguineous capillaries for them, they have previously thrown very fine injections into the arteries.

### V. On the Blennogenous Apparatus—the Source of the Mucous Matter.*

In order to examine this apparatus properly, it is necessary to select recent skin, injected and reddened with its blood. If the derma is white, either naturally or by maceration, nothing can be learnt from it.

The mucous matter of the skin becomes blended, soon after its secretion, with colouring matter, which occasions the various tints of horn, hair, scales, feathers, and so forth. The mucous matter and the horny matter are, as has already been observed, the same, the horny being originally mucous.

On examining the skin from within outwards, we find—

1. In the derma—

*a*. A *Blennogenous* apparatus, composed of a secretory gland, and of a canal which excretes the secreted matter, the mucus, which becomes horny matter by desiccation.

*b*. A *Chromatogenous* apparatus, composed of a secreting parenchyma, and of canals which excrete the product of secretion (squamous corpuscles).

2. External to the derma, and the joint effect of both these apparatuses—

*a*. The horny matter, or epidermis.

*b*. The hair, feathers, horns, hoofs, &c.

1. *The Blennogenous Apparatus.*—At the base of the derma we may distinguish small reddish glands, which, under the microscope or a common lens, appear knotted, irregular, and grooved by blood-vessels. They are enveloped in loose cellular membrane, and surrounded by small, transparent, adipose vesicles. From the summit of each of these glands there issues a canal or tube, which traverses the derma, and opens at the bottom of the furrows remarked on it. The tube is invested by a prolongation of the thin cellular membrane which envelops the gland. Capillary vessels or filaments may be seen adhering to the tube and to the glandular organ, into which our authors have seen a vessel of some size enter. The canals usually form a regular colonnade in the thickness of the derma. Sometimes

* Blennogenous, from βλεννα, mucus, and γεννάω, to produce.

the glands are placed at unequal heights, and appear to communicate with one another by means of intermediate canals. The ranges of excreting canals correspond to the furrows in the derma; they are perpendicular to the plane of the parenchyma secreting the colouring matter, to which we shall next direct attention.

2. *The Chromatogenous Apparatus.**—This is situated on the external surface of the derma, at the bottom of the furrows seen upon it, below and between the rows of papillary eminences. Superficially it gives rise to a great number of short excreting tubes, which end and pour out a peculiar matter at the bottom of the furrows. On its deep surface it is in relation with capillary vessels, and with the excreting canals of the blennogenous glands.

Its structure is areolar, spongy, and tough. The parenchyma itself, as well as its excreting canals, easily redden on account of their vascularity. Here the arterial system stops; this parenchyma forming, in the natural condition of the body, its limit, though the vessels of the papillæ, strictly speaking, do advance a little farther.

If we lacerate this tissue, we find in it innumerable little filaments, from which escape scales or colourless corpuscles in abundance. This is the only reservoir of such scales in the derma, and this parenchymato-glandular tissue may therefore be regarded as a peculiar organ, peculiarly constructed, penetrated by arterial and venous capillaries, and giving rise to excreting canals. The latter terminating at the same point as those of the blennogenous gland, pour into the mucus formed by this gland granules of pigment or colouring matter.

3. *Excreted Products of these Apparatuses.*—The joint product of the blennogenous and chromatogenous apparatus is the epidermis, or the horny matter. Our authors examine it as it occurs in the heel.

The inferior or deep surface of the entire epidermis present inequalities which correspond to the inequalities on the external surface of the derma. It exhibits, indeed, a cast of the latter. This surface of the epidermis has received the name of the reticular web of Malpighi. Two divisions (cloisons) may be distinguished in it. One fills the furrows of the derma, and adheres to it by prolongations which issue from the excreting blennogenous and chromatogenous canals. By this it is that the horny tissue is produced and renewed. When we attempt to separate the horny matter from the derma, we always experience a certain degree of resistance from this attachment to the furrows of the derma. Sometimes we may perceive the sort of roots sent into them, but, more commonly, they are indistinguishable, the horny matter coming off cleanly, as if only laid in the furrows. On the sides we may observe small holes, which give passage to the lymphatic vessels.

The other division of the horny layer, named by our authors inter-papillary, occupies the interval left by the bifid papillæ, and is prolonged into the infundibuliform depressions around the sudoriferous and inhalent canals. We may remark on the edges of these ridges of the horny matter, a lacerated appearance, occasioned by loose fragments of the sudoriferous canals.

---

* Chromatogenous, from κρῶμα, colour, and γεννάω, to produce.

On the right and left may be found the holes and sort of sheaths into which the papillæ penetrate obliquely.

On the external surface of the epidermis there are visible prominent lines, slightly concentric or parallel, and separated by grooves. Examined with a lens, these lines present alternately small papillary eminences and fissures, or slight depressions, containing the orifices of the hidrophorous canals. A line contains usually from four to six. It may easily be ascertained that the prominent lines have an imbricated disposition, so that in the motions of contraction, more particularly of the hand, they advance over one another, like the scales of a fish or of a serpent, whilst in the motions of extension they separate and lay open the bottom of the grooves. This imbricated appearance is very manifest where the skin forms folds, as at the bend of the elbow, the groin, &c.

The horny matter in man is whitish, elastic, transparent, and highly hygrometric. Its examination is extremely difficult, for it gives under the scalpel, like caoutchouc; when soft, it swells, and nothing can be made out of it, and when dry, it scales off at the least touch or pressure.

The epidermis of the whale is more adapted for anatomical study. The horny matter is secreted by a special apparatus, and organized like false membranes. It seems then to deserve the designation of tissue, accorded to it by Bichat.

The *epidermic tissue*, then, of the *whale** is smooth, spongy, and generally of a deep slate colour. Proceeding from without inwards, there may be detected in it with the naked eye two layers—one external, and parallel to the plane of the derma; the other consisting of straight fibres, extending perpendicularly between the derma and the external layer. We may see through this dark tissue the summits of the white nervous papillæ enveloped in their sheaths. The internal surface is dotted with apertures for the passage of the little papillary cones.

The respective thickness of the two layers is as follows: that of the external or horizontal layer, one line; that of the deep or perpendicular layer, three lines. As the thickness of the derma on the head of the animal is ten lines, the diameter of the whole skin is about fourteen lines.

In order to analyse the epidermic tissue, we must take a perpendicular fibre, and place it under a magnifier, on a slightly moistened glass.

The tissue is thus found to be composed of small scaly imbricated bodies, on a fine cellular web. These scales are readily detached, and stain water black under the appearance of granulations. A fibre is formed of a series of these scales inserted one into the other. The fibre is elastic and resistant.

The origin of the horny matter may be well seen in the whale, on account of its black colour. It fills all the space unoccupied by the papillæ. The black matter is excreted a little prior to its appearance on the outside of the derma, about half a line internal to which point we find it enclosed in a capsule or dermic membrane, at the bottom of which may be remarked little whitish or filamentous projections, which the capsule embraces; these are the excreting canals of the chromatogenous parenchyma.

---

* Again we must observe, that epidermis, in our authors' phraseology, signifies all the horny matter above the derma. Cuticle is only *its* external layer.

The horny matter is formed within, and has then almost a mucous consistence. It is protruded from within outwards, and pushes before it the previously-formed layers, which gradually solidify.

Our authors hang a good many observations on these facts; but we may waive the consideration of them, and having ascertained so much of the horny matter of the whale, return to its examination in man.

In order to examine the *horny matter in man,* the best plan is to place under a magnifier, in a little water, a portion of the most external epidermis, or of the mucus which is formed on the surface of the derma. If we then separate its component parts with the point of a scalpel, we may observe floating, in the midst of the fragments of inhalent vessels and sudoriferous canals, a number of apparently shapeless corpuscles. They are not really amorphous, this appearance depending on the violence employed either breaking them up, or leaving them partially agglutinated. In general, the scales affect the form of an irregular trapezium, are of a certain thickness, white, transparent, more or less striated, and disposed on a very fine areolar web, in an imbricated order. We may easily recognize, say our authors, in the scales, the product of the chromatogenous or colouring organ; and, in the pellucid web which supports them, the mucus of the glandular blennogenous organ.

The horny matter, excreted in the first instance in a fluid mucous state, moulds itself layer by layer on the papillæ, and envelops and protects the sudoriferous canals and the inhalent vessels, acquiring greater density as it becomes placed externally.

The horny tissue in the negro is black every where, excepting on the palm of the hands and the sole of the feet, where there are only slight traces of that colour. Its structure is the same as in the white race of men. On the heel of the negro the scales are colourless. In the rest of the body, the skin, when examined by a magnifier, is not so black as it appears to the naked eye. Over the papillæ it is white, in consequence of the white nervous matter shewing through it. The areolar web which supports the scales is always white.

### VI. On the Chromatogenous Apparatus, or Organs of the Secretion and Excretion of the Colouring Matter.

The mucous rete of Malpighi has been regarded as the sole seat of the colouring matter, which was said to be secreted by a vascular network, and collected and preserved in a semi-fluid form. Our authors adopt and present a very different view of it.

They have observed that if the skin is black or white, the free border of the scales is coloured black or white. The attached part of the scales and the cellular web into which it is implanted are always white, as are the parts accidentally contained in the epidermis, the nervous papillæ, the sudoriferous canals, and the inhalents. The scales then are the only parts in which colour resides. They were led to compare this with the wings of the lepidoptera, and to examine the colouration of flowers. They discover and dwell on both as analogical cases; but we may pass these considerations by.

Their observations on the colouring organs in man are the following:—

1. They presume that the form of the scale plays some part in the production of the phenomenon. Have the negro and the cetacea, or should they have, a scale of similar form? That of the European is trapezoid. The little articulated pieces which compose the petals differ according to the colour they present and the kind of flower to which they belong.

2. The scale is in more or less intimate communication through its pedicle with its secretory organ, and is nourished by a true circulation. It may therefore be considered as acting in a special manner on the fluid which is in contact with the pedicle by means of the areolar web to which it is attached.

It is certain that the colours are arranged with art in little compartments, so as to produce their optical effect. Our authors *presume* that the scale of the Negro is different in form from that of the European. Of course it ought to be so, to sustain their speculations. This consideration makes us wonder greatly that they have not found it so; for, really, they have seen so much and so minutely that they might as well have seen this too. Indeed it is inexplicable, if they can determine the exact form of the scale in the white man, in which the corpus mucosum itself is seen with comparative difficulty, why they cannot determine the exact form in the black man. They have shewn, we think, a little squeamishness in this instance. The colouring matter itself they admit to be formed by the glandular parenchyma to which reference has been previously made.

Thus the epidermis is not mere inorganic matter, or mucus mechanically expelled, but a tissue of complicated organization. It is connected with the important functions of exhalation and absorption, by the property which it possesses of suffering penetration by liquids; and this imbibition or endosmosis would seem to be the rudiment, or simplest degree of absorption in cutaneous and mucous surfaces.

The surface of the skin is marked with lines affecting geometrical figures, which have a fixed relation to the motions of the particular part. Thus, on the pulp of the fingers they form concentric circles—sinuosities in the palm of the hand—lozenge-shaped figures on the wrist, &c. These forms are such as are best adapted for the movements of the parts where they occur. As all the organs placed on the surface of the derma have an oblique direction, the disposition of all the epidermic coverings must of necessity be imbricated. We may remark this in the mode of implantation of the hairs.

If we look back on the anatomy of the skin, we may give the following brief sketch of its functions.

1. The blood carried by the arterial capillaries to the parenchyma that secretes the sweat, and returned from it by the veins, gives rise to the sensible and insensible perspiration.

2. Inhalents imbibe on the surface of the derma and in the epidermis extraneous fluids, and pour them into the lymphatics and the veins.

3. Nerves placed like sentinels on the surface of the body receive the impressions of touch.

4. Horny matter is secreted and moulded around the papillæ and the inhalent and sudoriferous canals. It is part of the apparatus of touch, a means of ornament and of defence, and eminently hygrometric. By virtue of the latter property it is penetrable more or less, according to its density, by the fluids with which it is brought into contact, and so performs an important part in the function of absorption or imbibition, which it regulates.

5. The derma is the tissue which supports, insulates, and protects the delicate instruments of these various functions.

A short section, or, rather, a few observations are devoted to the Pathology of the Skin. They are not of sufficient importance to detain us. Nor need we enter on the critical exposé of what other authors have written. All that we shall add before quitting the subject are the conclusions of our authors.

They believe they have shewed that:—

1. There exists an apparatus of exhalation, composed of hidrophorous or sudoriferous canals, which have a spiral disposition, and open on the surface of the skin by one extremity, while their other extremity is in connexion with a parenchymatous or glandular body, the diapnogenous apparatus, in the derma.

2. The inhalent canals are situated in the mucous body; they appear to be unprovided with orifices at their external extremity.

3. The medium in which these exhalent canals are disseminated is on the external surface of the derma.

4. The mucous matter, which by its induration forms the different epidermic layers, is the product of a particular apparatus; this is composed of a principal organ comparable to a gland and deeply placed in the derma, and of an excreting canal. The whole constitutes the blennogenous apparatus.

5. The epidermis or horny tissue resulting from this secretion and from its mixture with colouring matter, is traversed by the sudoriferous canals. The inhalent canals and the nervous papillæ enter it, but do not open externally.

6. A second apparatus, situated near the superficies of the derma, is devoted to the secretion of the colouring matter or pigment. This apparatus is composed also of minute glands and excreting canals. It constitutes the chromatogenous apparatus.

7. The matter secreted by the latter apparatus is mingled with the horny matter and its dependencies, and colours it.

8. The epidermis resulting from the secretion of the mucous or horny matter, and its mixture with the colouring matter, is disposed in successive layers. From this disposition results the scales of the superficial layer, or the *epidermis* of most authors.

9. The apparatus of sensation in the skin, is composed of papillæ or conoidal eminences essentially formed by the extremities of nerves, enveloped in epidermic layers. The nervous filaments arriving at these new sheaths, throw aside their neurilema, and terminate by anastomosing with one another in order to form arches.

10. A blood vessel, very inferior in size to the nerves, penetrates the papilla.

11. The nervous filaments, although they lose their neurilema when they enter the epidermic sheaths, still preserve a proper membrane.

12. The derma is a fibrous and vascular web, which contains the organs of secretion, and the commencement of their excreting canals, the origin of the exhalent canals, and many lymphatic and sanguineous vessels. The latter are chiefly found on the two surfaces of the derma especially upon its inner, and form numerous networks, constituting a sort of erectile tissue. The blood-vessels do not penetrate the mucous body, and beyond the derma we only observe them in the papillæ, where they are delicate, not numerous, and difficult to be distinguished. We may observe, however, by the aid of injection and of magnifying-glasses, lymphatic vessels on the external surface of the derma, in the deepest layers of the mucous body, and around the papillæ. They are arranged in net-works, rather close; their terminating apertures cannot be recognized.

This closes the memoir before us. It is the first of three, and the only one which has yet appeared. The second will be appropriated to the description of the accessory parts of the skin—hair, wool, feathers, scales, nails, horns, follicles, &c. The third will be occupied with the structure of the mucous membranes, and with physiological observations on the functions of them and of the skin.

It is not necessary for us to pronounce an opinion on this work. None can deny to its authors the merit of great labour, research, and ingenuity. Whether their statements are strictly accurate—whether they have seen more or less than nature offers, we do not feel ourselves competent to determine. Those only can pronounce an opinion, who have subjected the skin to very close microscopical observation, which we have not done.

